# Antagonistic Gcn5-Hda1 interactions revealed by mutations to the Anaphase Promoting Complex in yeast

**DOI:** 10.1186/1747-1028-6-13

**Published:** 2011-06-08

**Authors:** Azharul Islam, Emma L Turner, Johannes Menzel, Mackenzie E Malo, Troy AA Harkness

**Affiliations:** 1 Department of Anatomy and Cell Biology, University of Saskatchewan, Saskatoon, SK, S7N 5E5, Canada

## Abstract

**Background:**

Histone post-translational modifications are critical for gene expression and cell viability. A broad spectrum of histone lysine residues have been identified in yeast that are targeted by a variety of modifying enzymes. However, the regulation and interaction of these enzymes remains relatively uncharacterized. Previously we demonstrated that deletion of either the histone acetyltransferase (HAT) *GCN5 *or the histone deacetylase (HDAC) *HDA1 *exacerbated the temperature sensitive (*ts*) mutant phenotype of the Anaphase Promoting Complex (APC) *apc5^CA ^*allele. Here, the *apc5^CA ^*mutant background is used to study a previously uncharacterized functional antagonistic genetic interaction between Gcn5 and Hda1 that is not detected in *APC5 *cells.

**Results:**

Using Northerns, Westerns, reverse transcriptase PCR (rtPCR), chromatin immunoprecipitation (ChIP), and mutant phenotype suppression analysis, we observed that Hda1 and Gcn5 appear to compete for recruitment to promoters. We observed that the presence of Hda1 can partially occlude the binding of Gcn5 to the same promoter. Occlusion of Gcn5 recruitment to these promoters involved Hda1 and Tup1. Using sequential ChIP we show that Hda1 and Tup1 likely form complexes at these promoters, and that complex formation can be increased by deleting *GCN5*.

**Conclusions:**

Our data suggests large Gcn5 and Hda1 containing complexes may compete for space on promoters that utilize the Ssn6/Tup1 repressor complex. We predict that in *apc5^CA ^*cells the accumulation of an APC target may compensate for the loss of both *GCN5 *and *HDA1*.

## Background

Eukaryotic genetic information is packaged into chromatin, a highly organized and dynamic protein-DNA complex. The fundamental unit of chromatin, the nucleosome, is an octameric structure composed of two copies of each of the four core histones (an H3/H4 tetramer and two H2A/H2B dimers), surrounded by approximately 146 bp of DNA [1,2]. Many cellular processes depend on modifications of both DNA and histones within nucleosomes [3,4]. Modification of chromatin by histone acetyltransferases (HATs) and histone deacetylases (HDACs) play key roles in transcriptional regulation [5-9]. Post-translational acetylation of the highly conserved lysines within the N-terminal tail domains of the core histones is strongly correlated with transcriptional activation [5,10]. Although the precise mechanisms by which histone acetylation alters transcription are poorly understood [9-12], there is tremendous pressure to understand these mechanisms, as impaired histone modification is linked to many disease states [13].

The study of HAT and HDAC recruitment to promoters and their interaction with activators and repressors are essential for a better understanding of gene regulation. HATs and HDACs modify histones enzymatically throughout the genome [14]. Histone acetylation potentially regulates transcription by manipulating the higher-order folding properties of the chromatin fiber [15-17]. General control nonderepressible 5 (Gcn5) [18] was the first identified HAT and exists as the catalytic subunit in multiple high molecular weight complexes in yeast, including SAGA (Spt-Ada-Gcn5-Acetyltransferase), SLIK (SAGA-like), ADA (transcriptional ADAaptor), and the smaller HAT-A2 complex [19-23]. As part of the evolutionarily conserved SAGA complex, Gcn5 predominantly acetylates nucleosomal H3 lysines K9, K18, and K27 [24]. Defects in human SAGA subunits are associated with multiple disorders, including neurological diseases and aggressive cancers [25,26]. Gcn5 is a direct target for recruitment by transcriptional activators *in vitro *[27,28] and *in vivo *[29], which results in the acetylation of nearby histones [10]. Elongation of the transcripts initiated by Gcn5-containing complexes is carried out by the Elongator complex, which utilizes Elp3 as its primary HAT [30,31]. Cell cycle specific roles for Gcn5 have been reported, as recruitment of Gcn5 to a set of genes that are expressed in late mitosis requires SWI/SNF remodelling activity [32]. Furthermore, Gcn5 displays an overlapping pattern of localization with several HDACs [24,33,34]. Acetylation microarrays have shown that Rpd3 and Hda1 are the principal HDACs in yeast, affecting numerous promoters throughout the genome with little overlap between promoters [10,35]. Hda1, an evolutionary conserved HDAC, which deacetylates mainly histones H2B and H3 [36,37], is recruited to promoters via utilization of different Tup1/Ssn6 domains [38-40], resulting in local deacetylation. HDAC recruitment may form a positive feedback loop to repress transcription locally and facilitate the spreading of Tup1 into adjacent regions [41]. Tup1-mediated repression requires the deacetylation of histones within promoters [42-44], which may require direct recruitment of HDACs [36,45,46]. Overall, the mechanisms of Tup1/Ssn6-mediated transcriptional repression can be classified into 3 classes: (i) direct interaction with the activator; (ii) repression by changing chromatin structure; and (iii) interaction with the general transcription machinery [47,48]. It appears that different groups of genes have developed different strategies to utilize Tup1/Ssn6, enabling it to function as a global repressor.

Our work has linked the Anaphase Promoting Complex (APC), an evolutionarily conserved 13 subunit complex in yeast that is critical for mitotic progression and G1 maintenance [49-52], with chromatin assembly and histone acetylation through genetic interactions with chromatin assembly factor (CAF), HAT and HDAC mutants [53-57]. The APC is a ubiquitin-protein ligase (E3) that targets proteins that block the initiation of anaphase (Pds1) and mitotic exit (Clb2) for degradation. Various regulators govern APC activity in positive and negative manners, from phosphorylation and transcriptional control of APC subunits, to sequestration of APC activators [58-63]. For example, protein kinase A (a complex of Bcy1, Tpk1, Tpk2 and Tpk3) and Mad2 inhibit APC activity through phosphorylation and subunit sequestration, respectively. Activating phosphorylation is supplied by the polo-like kinase (Cdc5) and Cdc28. Furthermore, Cdc20, inhibited by a Mad2-dependent mechanism, binds and activates the APC to promote the metaphase/anaphase transition, while Cdh1, another APC-binding partner, drives APC-dependent mitotic exit. Previous studies by our group have expanded the APC's functional repertoire by showing that the mutant APC subunit allele, *apc5^CA ^*[54], genetically interacted with deletions of the HAT encoding genes *GCN5 *and *ELP3 *[57]. Strains harboring the *apc5^CA ^gcn5*Δ or the *apc5^CA ^elp3*Δ mutations had severely restricted growth at elevated temperatures compared to the single mutants. This interaction implies that the APC and these HATs positively interact, but a negative feedback loop appears apparent, as G1-specific Gcn5 instability was reduced in APC mutant cells. An additional synergistic genetic interaction between *hda1*Δ and *apc5^CA ^*was also observed, suggesting that the APC interacts positively with the HDAC Hda1 [57]. The study presented here focuses on a novel antagonistic relationship between *gcn5*Δ and *hda1*Δ that is revealed in *apc5^CA^*, but not *APC5 *cells. We provide further evidence that the APC works with multiple histone modifiers to drive cell cycle progression.

## Results

### *gcn5*Δ/*hda1*Δ interactions revealed in an APC mutant background

**Figure 1:**
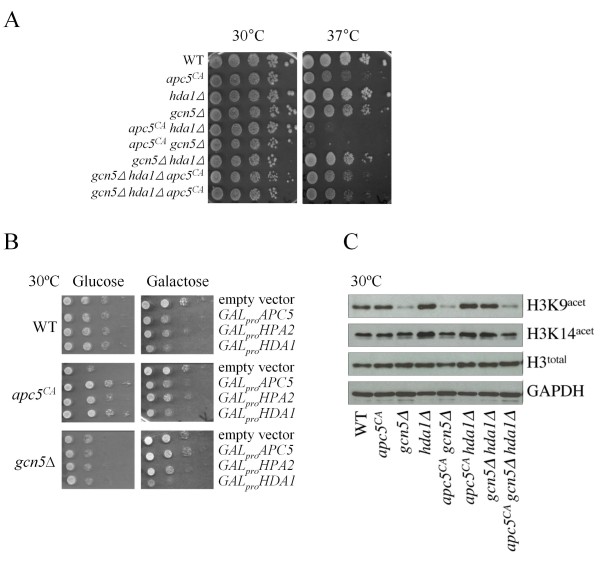
**Mutation to the APC subunit Apc5 reveals antagonistic interactions between Gcn5 and Hda1**. (A) Serial 10-fold dilutions of each strain were spotted onto YPD plates from left to right and incubated at the temperatures shown. (B) Serial dilutions using strains expressing the indicated plasmids were spotted onto SD-ura plates containing either 2% glucose or 2% galactose, and grown at 30°C for 2 and 3 days, respectively. (C) Protein lysates were prepared from the mutants shown and characterized by Westerns using the antibodies indicated. Antibodies against GAPDH were used as load controls.

In a recent screen, we identified HAT (*gcn5*Δ) and HDAC (*hda1*Δ) deletions that severely impacted the *apc5^CA ^*(chromatin assembly defective) [54,57] temperature sensitive (*ts*) phenotype, indicating that both proteins have a positive influence on Anaphase Promoting Complex (APC) activity. The *apc5^CA ^*mutation was identified in a chromatin assembly mutant screen; the allele contains an AT deletion altering amino acid 12, which created an in-frame stop codon 12 amino acids further along [54]. We recently observed that the apc5^CA^-TAP (Tandem Affinity Protein purification) protein migrates faster, with less intensity, than the wild type Apc5-TAP by SDS-PAGE, indicating that *apc5^CA ^*encodes an N-terminal truncation (data not shown). Here we show that deletion of *HDA1 *in *gcn5*Δ cells had no apparent effect (Figure 1A), whereas deletion of *HDA1 *in *apc5*^*CA *^*gcn5*Δ cells improved *ts *growth. The *apc5^CA ^*background therefore allowed the study of a previously uncharacterized antagonistic interaction in yeast between Gcn5 and Hda1. Plant GCN5 was also found to interact antagonistically with HD1, the Hda1 orthologue, to regulate light-responsive gene expression [64], but mechanisms remained undetermined.

To examine whether Hda1 positively interacted with the APC, we expressed galactose driven *HDA1 *carrying a C-terminal HA tag (*GAL_pro_HDA1-HA*) at low levels in WT, *apc5^CA ^*and *gcn5*Δ cells by using glucose as a carbon source (Figure 1B). Recently, we observed that mRNA levels of *GAL_pro_GCN5-HA *were elevated 100-fold when grown on 2% glucose and 900-fold when grown on 2% galactose [57]. However, Gcn5-HA protein expression remained low even though *GCN5-HA *mRNA was 100-fold elevated when grown on 2% glucose. As shown with *GCN5 *[57], low-level *GAL_pro_HDA1-HA *expression improved *apc5^CA ^*growth (Figure 1B). This is not necessarily a general feature of histone modifying proteins, as deletion or overexpression of the HAT *HPA2 *had little effect on *apc5^CA ^*cells (Figure 1B) [57]. Although the yeast Hpa2 has not yet been shown to acetylate histones *in vivo*, a bacterial acetyltransferase that does acetylate eukaryotic histones is most closely related to Hpa2, and Hpa2 does acetylate H3 *in vitro *[65,66]. Moreover, Hpa2 appears to be active, as overexpression reduces growth of *gcn5*Δ cells, whereas expression on glucose improves growth of *apc5^CA ^*cells (Figure 1B).

A further connection between Gcn5 and Apc5 was observed by the rescue of *GAL_pro_APC5-HA *overexpression toxicity by deletion of *GCN5 *(Figure 1B). It is unlikely that Apc5 protein levels induced from the *GAL *promoter are compromised in *gcn5*Δ cells, as expression of *HPA2 *and *HDA1 *from the *GAL *promoter reduces *gcn5*Δ growth. Overexpression of *APC5 *from the *CUP1 *promoter also reduced yeast replicative lifespan [60]. Rescue of *APC5 *toxicity by *GCN5 *deletion is consistent with our recently proposed hypothesis that Gcn5 is required for APC activity, and may provide an explanation as to why *GCN5 *[57] and *HDA1 *(Figure 1B) overexpression is toxic, considering that overabundance of Apc5 is detrimental to cells.

Next, we asked whether mutations to *APC5 *influenced acetylation of histone H3 lysine 9 or 14 (H3K9/14) in *gcn5*Δ and *hda1*Δ cells. Gcn5 appears to play a greater role on H3K9, compared to H3K14, whereas loss of *HDA1 *results in increased acetylation of both H3K9 and H3K14 (Figure 1C). The *apc5^CA ^*background did not change the acetylation status of H3K9/14 in *gcn5*Δ or *hda1*Δ cells, suggesting the *apc5^CA ^*background may be revealing an effect other than global histone H3 acetylation. H3K9Ac was reduced in *gcn5*Δ, *apc5^CA ^gcn5 *Δ and *apc5^CA ^gcn5*Δ *hda1*Δ cells, but not in *gcn5*Δ *hda1*Δ cells. The ability to acetylate H3K9 in *gcn5*Δ *hda1*Δ cells indicates that on a global level, other HATs can use H3K9 as a substrate. However, at the gene level, deletion of *GCN5 *was previously shown to reverse histone hyperacetylation at the *PHO5 *promoter when *HDA1 *was deleted [67]. Therefore, we tested whether transcript levels are influenced by *apc5^CA ^*in *gcn5*Δ or *hda1*Δ cells.

### The *apc5^CA ^*allele increases transcript levels in *hda1*Δ cells

**Figure 2:**
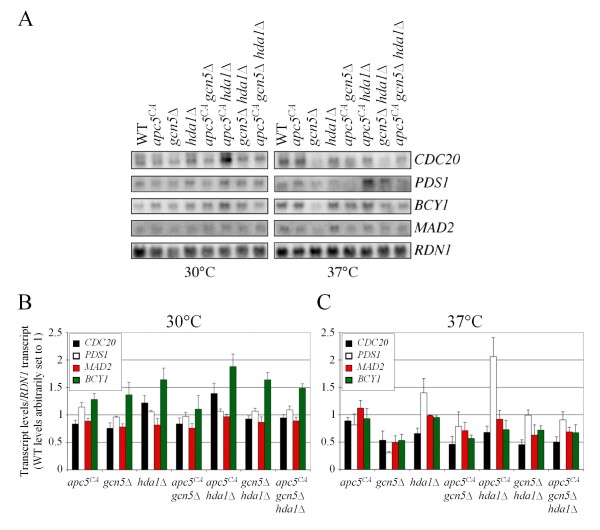
**Expression of *PDS1*, an APC antagonist, is specifically elevated in *apc5^CA ^hda1*Δ cells at 37°C**. (A) Northern analyses were conducted on total RNAs extracted from the various mutants and probed with sequences derived from the coding regions of the genes indicated. *RDN37-2 *(*RDN1*) was used as a loading control. All bands from the 30°C experiments (B) and the 37°C experiments (C) were quantified by ImageJ and normalized to the *RDN1 *signal. Densitometry was performed on two Northern experiments and two reverse transcriptase experiments. The data was combined and the means and standard errors were plotted.

Since the *apc5^CA ^*allele had little effect on global histone H3K9/14 acetylation, we asked whether individual gene transcripts were altered. We chose to study several genes involved in APC function, as altered expression of APC regulators may underlie the observed growth phenotypes. Thus, we performed Northern and reverse transcriptase PCR (rtPCR) experiments to determine expression of *CDC20*, *PDS1*, *BCY1 *and *MAD2*. Cdc20 plays a positive role in APC activity, whereas Pds1, Bcy1 and Mad2 have a negative impact [58-63]. Northerns (Figure 2A) and rtPCR (data not shown) both show that compared to *RDN1*, expression of the tested transcripts were reduced in *gcn5*Δ cells, especially at 37°C, whereas transcripts in *hda1*Δ cells were relatively unimpaired. The bands from 2 Northerns and 2 rtPCR experiments were scanned, quantified and averaged, with the expression of each gene for each experiment normalized to *RDN1*. This number is relative to expression in the wild type strain, which was set to 1 (Figures 2B, C). Although previous microarray analyses in *gcn5*Δ and *hda1*Δ cells did not identify these genes [68,69], the approximate 2-fold decrease in transcript levels in *gcn5*Δ cells (Figure 2C) suggests Gcn5 is involved in expression of the tested genes. However, while the *apc5^CA ^*allele had no apparent effect on transcript levels in *gcn5*Δ cells, in *apc5*^*CA *^*hda1*Δ cells, *BCY1 *transcripts (Figure 2B) and *PDS1 *transcripts (Figure 2C) were clearly elevated. The loss of this effect in the triple mutant suggests Gcn5 may be required for elevated transcription in *apc5^CA ^hda1*Δ cells.

### Increased *PDS1 *transcripts in *apc5*^*CA *^*hda1*Δ cells correlates with increased promoter acetylation

**Figure 3:**
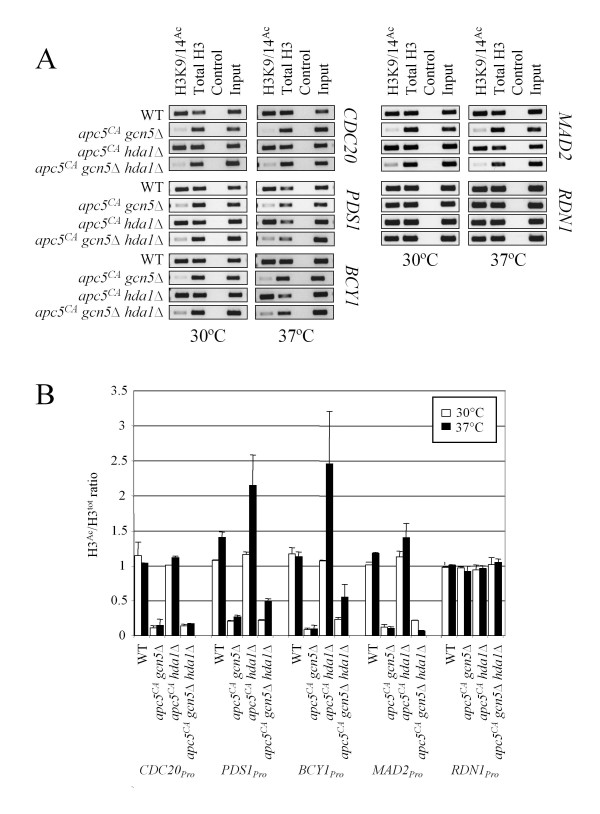
**Histone H3 acetylation at promoter regions is elevated specifically in *apc5^CA ^hda1*Δ cells at 37°C**. (A) ChIP was performed using lysates derived from the mutants shown and antibodies against total H3 or H3 acetylated at both K9 and K14. A mock treatment lacking antibody was used as a control. Once the crosslinks were reversed and DNA recovered, "end point" PCR was performed using primers against the genes shown that amplified 200 bp regions of the promoter. 10% of the reaction was used as input. (B) Two independent experiments were quantified, with the means and standard errors represented graphically, as previously described [66,70].

Our data suggests the *apc5^CA ^*allele enhances the transcript levels of some of the tested genes in *hda1*Δ cells. We next tested whether promoter acetylation of these genes was similarly impacted using chromatin immunoprecipitation (ChIP) with antibodies that recognized acetylated lysines 9 and 14 on histone H3 (H3K9/14^Ac^), and primers that amplified 200 basepair fragments immediately upstream of the transcriptional start site of the genes studied above. We used H3K9/K14^Ac ^antibodies to capture acetylation of both H3K9 and H3K14 as our studies show these residues are targeted by Gcn5 and Hda1. We assessed promoter acetylation in *gcn5*Δ, *hda1*Δ and *gcn5*Δ *hda*Δ*1 *mutants in the *apc5^CA ^*background (Figure 3A). Antibodies against total H3 and a no antibody mock treatment were used as controls. The bands in all experiments were quantified and analyzed (Figure 3B). Once background densities were subtracted from all bands, the H3K9/14^Ac^/total H3 ratio was determined. The values represent two independent experiments, as described previously [66,70]. H3 promoter acetylation was reduced in both *apc5*^*CA *^*gcn5*Δ and *apc5*^*CA *^*gcn5 hda1*Δ cells, similar to the transcript patterns at 37°C (Figure 2C), strengthening the notion that Gcn5 HAT activity is tightly correlated with transcription. However, it is interesting to note that while promoter acetylation is equally low in *apc5*^*CA *^*gcn5*Δ cells at 30 and 37°C, transcript defects are only obvious at 37°C. Notably, a previous study observed that Gcn5-dependent transcription and promoter histone acetylation activities could be uncoupled [71].

Consistent with our observations that transcript levels of *BCY1 *and *PDS1 *increase in *apc5^CA ^hda1*Δ cells, we detected increased *BCY1 *and *PDS1 *promoter acetylation in these cells, specifically at 37°C. Transcript levels and promoter acetylation are both increased with *PDS1 *at 37°C in *apc5*^*CA *^*hda1*Δ cells. However, we note some differences in the patterns observed. For example, *BCY1 *transcripts are not elevated in *apc5^CA ^hda1*Δ cells at 37°C while promoter acetylation is. This may reflect the complex nature of the factors assembled at promoters that is not addressed in this study.

### Gcn5 promoter occupancy increases in the absence of Hda1

**Figure 4:**
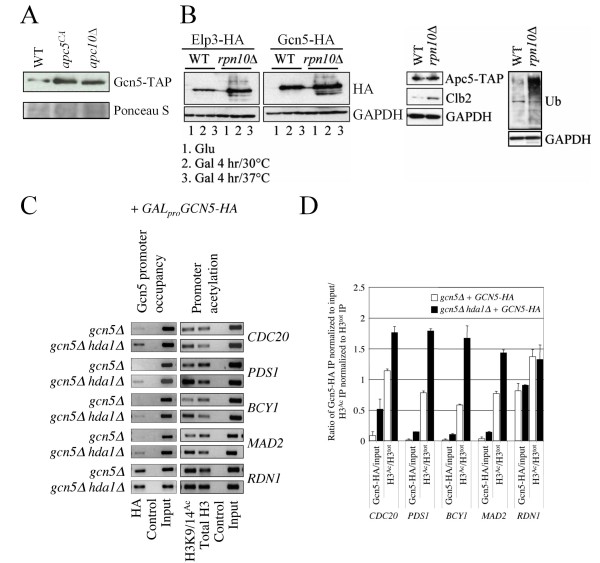
**Deletion of *HDA1 *results in increased Gcn5 at promoters**. (A) Steady-state Gcn5-TAP in different mutant backgrounds was determined in early log phase asynchronous cells grown at 30°C by Western blotting. Westerns were performed using antibody against TAP and the membrane was stained with Ponceau S to confirm equal protein load. (B) Plasmid borne HA-tagged *GCN5 *and *ELP3*, driven by the galactose inducible promoter, were expressed in cells lacking the proteasome ubiquitin receptor Rpn10. Cells were grown overnight in 2% glucose to early log phase. The glucose-supplemented media was washed away and the cells were resuspended in 2% galactose-supplemented media. The cells were then split with one half incubated at 37°C and the other half left at 30°C. The cells were incubated for an additional 4 hours, afterwhich proteins were harvested and analyzed with antibodies against HA or GAPDH as a load control. Controls for the experiment included endogenous *APC5-TAP *in *rpn10*Δ cells, as well as the detection of endogenous Clb2 and Ub in WT and *rpn10*Δ cells using commercially available antibodies. (C) Protein/DNA complexes were recovered from the mutants shown following *GAL*-induction using antibodies against either the HA epitope, total H3, or H3K9/14^Ac^. A mock treatment was conducted where antibody was omitted. Recovered DNA was used as template in "end point" PCR reactions using primers that amplified the promoter regions indicated. 10 μl of each reaction was separated by agarose gel electrophoresis and scanned. (D) Two independent experiments were performed. The gels were scanned and quantified using ImageJ. The means and standard errors were plotted.

One possible scenario to explain increased *PDS1 *promoter acetylation and transcription in *apc5*^*CA *^*hda1*Δ cells may be increased availability of Gcn5 or related HATs due to the *apc5^CA ^*allele. It was previously reported that in cells expressing defective *TUP1*, increased Gcn5 was observed at Tup1-repressible promoters, thereby derepressing transcription [40]. We have speculated that the APC may target Gcn5 for turnover in order to progress through the G1/S transition [57]. To examine this possibility, endogenous *GCN5 *was TAP-tagged in WT, *apc5^CA ^*and *apc10*Δ cells and detected by Westerns in asynchronous early log phase cells. Gcn5 protein levels were indeed increased in both *apc5^CA ^*and *apc10*Δ cells (Figure 4A). While this does not explain the genetic interaction between *gcn5*Δ and *hda1*Δ in *apc5^CA ^*cells, it does suggest the possibility that a factor related to Gcn5 may also be elevated in *apc5^CA ^*cells. Consistent with this hypothesis, we observed increased Gcn5-HA and Elp3-HA, expressed from the *GAL *promoter, in cells lacking the proteasome ubiquitin receptor Rpn10 (Figure 4B). As controls, we TAP-tagged *APC5 *in *rpn10*Δ cells, which was unaffected by *rpn10*Δ. Furthermore, GAPDH was also unaffected by *rpn10*Δ, whereas ubiquitinated proteins did accumulate. Therefore, in cells lacking a functional ubiquitin system, at least Gcn5 and Elp3 accumulate.

Next we asked whether promoter occupancy by Gcn5 correlated with gene expression and promoter acetylation. *GAL_pro_GCN5-HA *was induced in *gcn5*Δ and *gcn5*Δ *hda1*Δ cells so that the only Gcn5 expressed was HA tagged. *gcn5*Δ cells expressing *GAL_pro_GCN5-HA *grew like WT (data not shown), and were considered the WT control for this experiment. ChIP was performed in lysates prepared from these cells. Control ChIPs were performed using untagged lysates (data not shown), and reactions without antibody, neither of which produced PCR products. Primers against the 5', middle, and 3' regions of *CDC20 *demonstrated that Gcn5-HA recruitment was most prominent at the promoter and was reduced 5' to 3' (data not shown). We found that in *HDA1 *cells expressing *GCN5-HA*, very little Gcn5-HA was present at the promoters tested compared with the *RDN1 *promoter (Figures 4C and 4D). In *hda1*Δ *GCN5-HA *cells, however, increased Gcn5-HA promoter recruitment was observed. The increases observed were slight except for the *CDC20 *promoter. Promoter acetylation also increased in *hda1*Δ cells, consistent with increased recruitment of Gcn5. These observations present the possibility that i) increased promoter H3K9/14 acetylation in *hda1*Δ cells is due to increased Gcn5-HA promoter recruitment; and/or ii) Hda1 may block access of Gcn5 to promoters.

**Figure 5:**
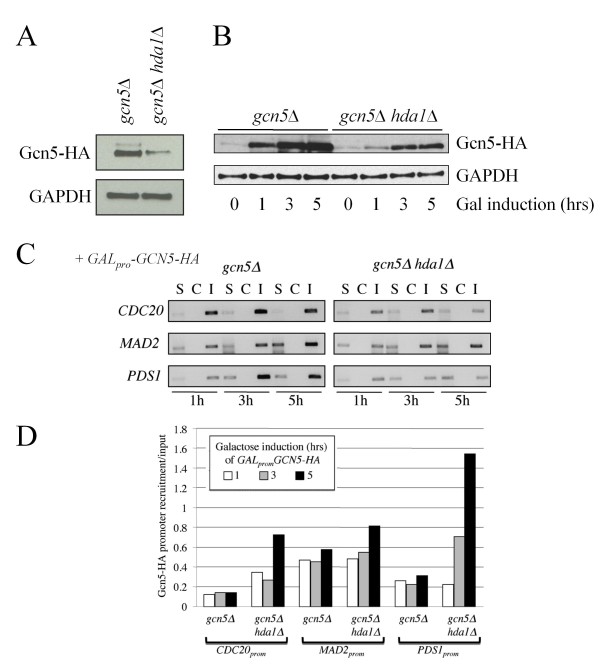
**Gcn5 promoter occupancy is kept at an equilibrium in WT cells, but increases over time in *hda1 *cells**. (A) Western analyses of Gcn5-HA expression in *gcn5*Δ and *gcn5*Δ *hda1*Δ cells following a 5 hour 4% galactose-induction. Antibodies against GAPDH were used as a load control. (B) A galactose-induction time-course was performed in *gcn5*Δ and *gcn5*Δ *hda1*Δ cells expressing *GAL_pro_-GCN5-HA*. Protein samples were removed at the times shown for Western analyses with antibodies against HA and GAPDH. (C) From the time-course described above, samples were also removed for ChIP. Recovered DNA was used as a template in "end point" PCR reactions. S, sample with antibody; C, control without antibody; I, 10% lysate input. (D) The gel in (C) was scanned, analyzed using ImageJ and plotted.

It is possible that increased Gcn5-HA recruitment is due to increased *GAL_pro_*-driven Gcn5 expression in *hda1*Δ cells, since Hda1 represses galactose-induced gene activation [72]. We assessed expression of Gcn5-HA in the strains used above and observed that *GAL_pro_GCN5-HA *expression after a 5 hour induction period was reduced in *hda1*Δ cells (Figure 5A). Therefore, it is unlikely that the decreased levels of *GAL*-promoter driven *GCN5 *in *hda1*Δ cells are due to Hda1's influence on the *GAL *promoter. Considering that *hda1*Δ cells express less *GCN5 *than WT, yet recruit a greater amount of Gcn5 to promoters, a much greater proportion of Gcn5-HA must be available for recruitment in *hda1*Δ cells. To examine this possibility, we performed ChIP using lysates prepared from *GAL_pro_GCN5-HA *expressing cells after 1, 3 and 5 hours of induction (Figure 5B). Gcn5-HA was recruited to each tested promoter (Figures 5C and 5D). When normalized to input, Gcn5-HA recruitment in *HDA1 *cells was similar at each induction timepoint (Figure 5D). In *hda1*Δ cells however, Gcn5-HA recruitment was again increased, and recruitment increased the longer the induction. Together, our data suggests that in the absence of Hda1, Gcn5-HA continually gains access to the tested promoters.

### Tup1 occludes Gcn5 promoter occupancy

**Figure 6:**
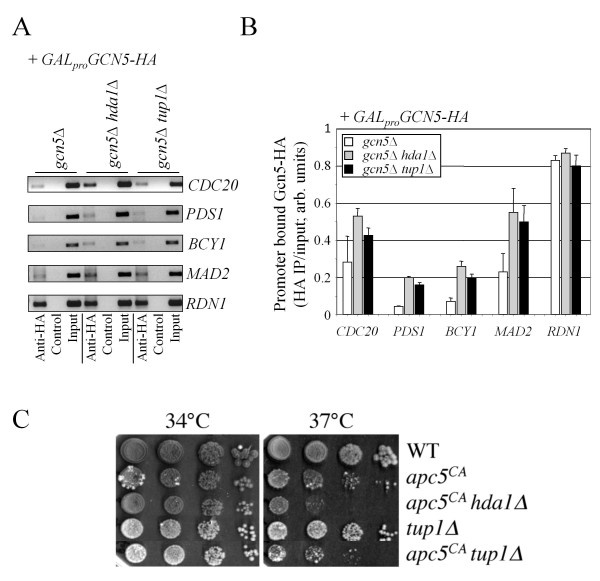
**Tup1 occludes Gcn5 recruitment**. A) ChIP was performed using the cells shown expressing *GAL_pro_-GCN5-HA *following a 5 hour galactose induction, as described above. (B) Two independent experiments were scanned and processed using ImageJ, with the means and standard errors shown. (C) Strains lacking *TUP1 *were constructed in WT and *apc5^CA ^*backgrounds. Growth phenotypes were assessed by spot-dilutions, followed by incubation at 34°C and 37°C.

We next tested whether the impact of Hda1 on Gcn5 promoter accessibility involved the corepressor complex Ssn6/Tup1. Several reports have demonstrated that the Ssn6/Tup1 corepressor utilizes Hda1 to repress transcription of target genes [36,41,68]. Furthermore, Tup1 has been shown to recruit Gcn5 to repressed promoters [73-75]. It was proposed that this may set the stage for derepression of silent genes. Thus, *GAL_pro_GCN5-HA *was induced in *hda1*Δ and *tup1*Δ cells as the only source of Gcn5, followed by ChIP. Gcn5-HA expression in *hda1*Δ cells was reduced compared to WT, but expression in *tup1*Δ cells was unchanged (data not shown). We found that in otherwise WT strains (*gcn5*Δ + *GALproGCN5-HA*), Gcn5-HA was weakly recruited to the tested promoters (Figures 6A and 6B). In strains lacking *HDA1 *or *TUP1*, Gcn5-HA promoter occupancy was observed to increase. We also observed that in cells lacking *SSN6*, promoter recruitment of Gcn5-HA increased (data not shown). These results suggest that Hda1 may work together with the Ssn6/Tup1 corepressor complex to impede access of Gcn5 to the tested promoters. However, Hda1 and Gcn5 may also compete for Tup1 interactions. It is also feasible that Tup1 utilizes different mechanisms to reduce Gcn5 promoter occupancy.

To distinguish between these possibilities, we predicted that if Tup1 and Hda1 work together, then deletion of *TUP1 *in *apc5^CA ^*cells should have the same synergistic effects as an *HDA1 *deletion. Our results show that deletion of *TUP1 *impairs the *apc5^CA ^*phenotype (Figure 6C), similar to an *hda1*Δ mutation. This suggests that both Hda1 and Tup1 perform a function that is beneficial to APC activity. However, it does not necessarily indicate they work together to perform this task.

### Hda1 and Tup1 likely interact at promoters, which can be inhibited by Gcn5

**Figure 7:**
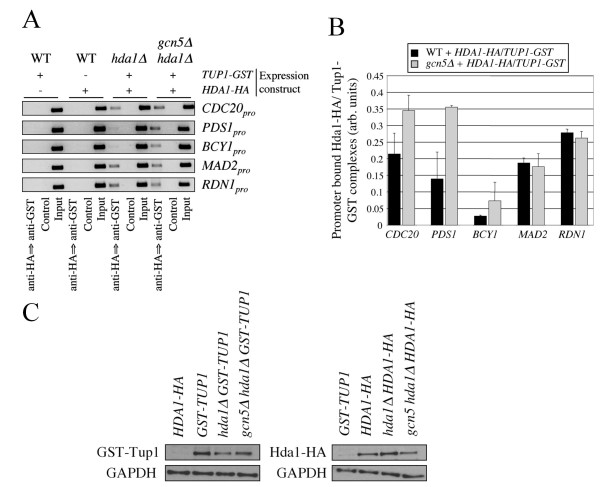
**Gcn5 can inhibit Hda1-Tup1 associations at some promoters**. (A) Sequential ChIP was used to observe Hda1-Tup1 physical interactions at specific promoters. WT, *hda1*Δ and *gcn5*Δ *hda1*Δ cells expressing combinations of *GAL_pro_-HDA1-HA *and *CUP1_pro_-TUP1-GST *were induced using 4% galactose for 5 hours and 0.4 mM CuSO_4 _for 3 hours. ChIP reactions were first performed with antibodies against HA. Bound proteins were eluted from beads using 10 mM DTT for 30 minutes at 37°C. The eluted proteins were then incubated with anti-GST antibodies. The immune complexes were isolated again, cross links were reversed, and "end point" PCR was performed using the recovered DNA as template. (B) Two independent experiments were performed and processed using ImageJ. The means and standard errors are shown. (C) Westerns showing expression of the proteins used in the sequential ChIP experiment.

Others have also shown Tup1 and Hda1 functionally interact to repress gene transcription [36,41,68], and to associate *in vitro *[36], but not necessarily *in vivo *[46]. To investigate whether Tup1 and Hda1 do function together, we asked if Tup1 and Hda1 can physically interact at promoters, and if Gcn5 can influence this. To do so we performed sequential ChIP in cells expressing a combination of Hda1-HA and/or GST-Tup1. ChIP was first performed using antibodies against HA. Bound proteins were released, recovered, and incubated with antibodies against GST. Bound protein/DNA complexes were again isolated and PCR was performed using primers against the test promoters. The results show that in cells expressing either Hda1-HA or GST-Tup1, no bound DNA was recovered (Figure 7A and 7B). However, in cells co-expressing the plasmids, PCR fragments were obtained for all promoters tested. This supports the idea that Tup1 and Hda1 can associate *in vivo *at specific promoters. Nonetheless, this could also reflect close, but independent Hda1 and Tup1 binding on the same promoter. In *gcn5*Δ cells co-expressing the plasmids, putative complex formation was again observed, and was visibly increased at *CDC20*, *PDS1*, and *BCY1 *promoters, suggesting Gcn5 may negatively impact this interaction. Figure 7C shows that the proteins were expressed similarly in the strains used. Our experiments do not differentiate between Hda1 and Tup1 physically binding, or whether they simply bind adjacent DNA sequences, but it is important to note Hda1 and Tup1 were previously shown to physically associate [36], and that the interaction observed by sequential ChIP is enhanced by *GCN5 *deletion.

**Figure 8:**
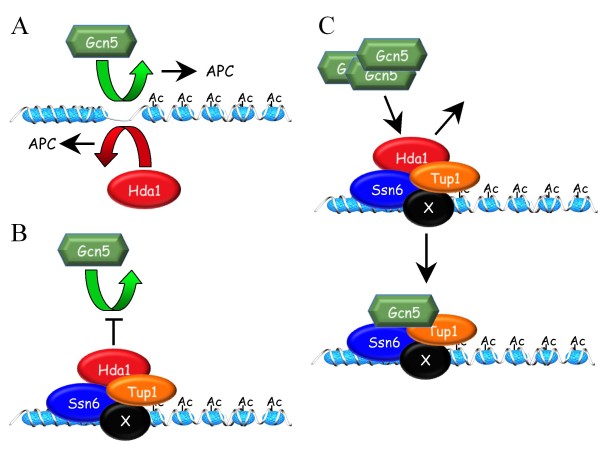
**A model depicting potential interactions between Gcn5 and Hda1**. (A) The HAT Gcn5 and the HDAC Hda1 have opposing functions that individually benefit APC function. (B) and (C) Gcn5 and Hda1 appear to compete for Tup1 binding. (B) If Hda1 first gains access to the promoter, recruitment of Gcn5 is partially blocked. (C) Under conditions where gene transcription must be derepressed, Tup1 may recruit Gcn5 to the promoter to prime transcriptional initiation, thus displacing Hda1. The protein labelled × represents a DNA binding factor that recruits the Tup1/Ssn6 corepressor complex to silent genes.

Taken together, the results presented in this report suggest a competitive interaction can occur between Hda1/Tup1 and Gcn5 at promoters (Figure 8). Our results suggest that the presence of Hda1/Tup1 (and likely Ssn6) occludes, at least partially, the recruitment of Gcn5 to some promoters. Gcn5, on the other hand, may impede Tup1-Hda1 interactions by competing for Tup1 binding. It is possible that the *gcn5*Δ/*hda1*Δ genetic interaction is prominent in *apc5^CA ^*cells due to the accumulation of an APC target, perhaps another HAT, capable of suppressing *gcn5 hda1***Δ **impairments.

## Discussion

### Novel Gcn5/Hda1 antagonistic functional interactions are revealed when APC activity is compromised

The work presented here provides evidence to support a model in which the HAT Gcn5 and the HDAC Hda1 functionally interact at promoters to determine transcriptional readouts (Figure 8). In otherwise WT cells, mutations to *GCN5 *or *HDA1 *do not create significant growth defects, whereas in *apc5^CA ^*cells, these same mutations produce severe *ts *growth defects (Figure 1A). The focus of this study was to characterize an antagonistic functional *gcn5*Δ/*hda1*Δ interaction revealed in the *apc5^CA ^*background, as the severe *apc5^CA ^gcn5*Δ and *apc5^CA ^hda1*Δ *ts *defects are suppressed in *apc5^CA ^gcn5*Δ *hda1*Δ cells. Growth phenotypes associated with deletion of *GCN5 *have been shown in two separate Synthetic Genetic Array (SGA) genome-wide screens to be suppressed by deletion of *HDA1 *[76,77]. However, spot dilution analysis of the *gcn5*Δ and *hda1*Δ cells on YPD did not reveal any phenotypes [76], as shown in our study (Figure 1A). Thus, the *gcn5*Δ *hda1*Δ antagonistic interaction is not apparent under normal growth conditions, such as on YPD, but under conditions imposed by the SGA screen (selective media, for example), the antagonistic interaction can be exposed. The influence of the *apc5^CA ^*allele on this interaction was investigated. The *apc5^CA ^*allele had little effect on global histone H3 acetylation status in *gcn5*Δ and *hda1*Δ cells, but did cause the increase of *BCY1 *and *PDS1 *transcripts in *hda1*Δ cells (Figures 1C, 3). Both Bcy1 and Pds1 proteins antagonize APC activity and may be involved in the enhanced growth defect when APC is mutated. Therefore, in *apc5^CA ^*cells, it may be the inappropriate expression of inhibitory transcripts that are paramount to synergistic *apc5*^*CA *^*gcn5*Δ and *apc5*^*CA *^*hda1*Δ phenotypes.

A molecular mechanism explaining the Gcn5/Hda1 interaction likely involves competition for Tup1 binding. We observed that in cells lacking *HDA1 *or *TUP1*, Gcn5 recruitment at our tested promoters was increased (Figures 4 and 6). On the other hand, deletion of *GCN5 *increased Hda1-Tup1 physical interactions at promoters (Figure 7). A competition between Hda1 and Gcn5 for Tup1 binding is a possibility worth considering, as both Hda1 and Gcn5 have been shown to physically interact with Tup1 [36,73-75]. However, in *gcn5 hda1*Δ cells this mechanism would not be possible. In addition to the accumulation of Gcn5 in *apc5^CA ^*cells, we observed that Elp3 also accumulates when the ubiquitin system is compromised (Figures 4A, B). We previously demonstrated that *gcn5*Δ and *elp3*Δ deletions impair *apc5^CA ^*defects, that *GCN5 *and *ELP3 *overexpression stalls the cell cycle in G1, and that Gcn5 G1-specific instability is reversed in APC mutants [57]. Thus, when *apc5^CA ^*is combined with* gcn5*Δ hda1Δ, an APC target likely accumulates that creates novel transcripts that allow bypass of the severe *ts *defects observed in the double mutants. Elp3 is an attractive candidate since it is involved in elongating transcripts initiated by Gcn5 containing complexes [31]. A global transcript analysis is likely required to follow this further. Our previous work suggests that the *apc5^CA ^*phenotype is sensitive to global transcript levels [57].

### Hda1-dependent occlusion of Gcn5 from promoters requires Tup1

Several reports describe the recruitment of the Tup1/Ssn6 repressor complex to DNA via interactions with multiple partners [41,48,68]. Once recruited, Tup1 then contacts H3 and H4 N-terminal tails [78]. Mechanisms employed to recruit Tup1/Ssn6 to promoters by the various individual interacting partners appears to be complex, seems to vary, and may have overlapping roles. Gcn5-HA recruitment to the tested promoters was increased in *hda1*Δ, *tup1*Δ and *ssn6*Δ cells (Figure 6; data not shown), indicating that the interaction of Hda1 with the Tup1/Ssn6 repressor complex is necessary to block access to Gcn5. Tup1 and Hda1 did indeed co-immunoprecipitate while bound to the same promoters, as shown by sequential ChIP (Figure 7). We find it unlikely that Tup1 and Hda1 are simply associating independently at adjacent sequences within the 200-basepair DNA PCR fragment, since they have been shown to interact previously [34], and are part of large complexes [19-23], but we cannot discount this possibility. However, we observed that in *gcn5*Δ cells, Hda1-Tup1 association increased at some promoters (*PDS1 *and *BCY1*), suggesting Gcn5 opposes complex formation. The mechanism of action that Gcn5 uses to block Hda1-Tup1 association remains unclear. Previous reports indicating that Tup1 is capable of recruiting and interacting with Gcn5/SAGA at promoters [73-75] suggest it is possible that Gcn5 and Hda1 may compete for Tup1 interaction. The scenario for recruiting either Gcn5 or Hda1 would differ, implying other proteins may be involved in deciding whether Gcn5 or Hda1 gain access. We were unable to observe complex formation between Gcn5-TAP and Hda1-HA in whole cell lysates (data not shown), indicating possible exchange of Gcn5 and Hda1 at Tup1 complexes does not require Gcn5-Hda1 association. It is also possible that Gcn5-Hda1 physical interactions are transient and promoter specific, therefore may not be detectable using the methods applied here. Nonetheless, support for our model was provided by reports describing recruitment of Gcn5 to promoters by the Tup1/Ssn6 complex under osmotic stress conditions [40,74], indicating that Tup1/Ssn6 may be a transcriptional activator under certain conditions.

## Conclusions

The results presented in this manuscript provide evidence for a complex network of interactions between a mitotic/G1 cell cycle regulator (the APC), and antagonistic interplay between a HAT (Gcn5), and an HDAC (Hda1). Gcn5 is known to function during mitosis [32,57,79,80]. Data on the role Hda1 plays in cell cycle progression is limited, but Hda1 may provide some function to ensure histones are deacetylated prior to passage through mitosis [81]. It is noteworthy that Gcn5 and Hda1 expression is temporally regulated during the cell cycle (microarray data compiled at *Saccharomyces *Genome Database), providing insight into how the potential competition for Tup1 binding could be regulated. APC mutations cause cell cycle progression to stall during mitosis, potentially skewing the equilibrium between Gcn5 and Hda1 promoter recruitment if the cell cycle does indeed influence Hda1 and Gcn5 recruitment. Future work will focus on identifying the molecular mechanisms regulating how cell cycle progression influences chromatin dynamics. Chromosome synthesis and segregation defects are widely associated with human disease, thus continued work into furthering our understanding of this process is vital.

## Methods

### Media, yeast strains, plasmids and general methods

Cells were grown in YPD (1% yeast extract, 2% peptone, 2% glucose) or synthetic complete drop-out media (SD; 0.17% yeast nitrogen base, 0.5% (NH_4_)_2_SO_4_, 2% glucose [or 4% galactose], 1.3 g amino acid drop-out powder/1 L, 1 tablet NaOH). Genes under the control of the galactose promoter were induced with 4% galactose for 5 hours. All yeast strains were S288c derivatives unless mentioned otherwise (Table 1). Double and triple mutants were created by crossing appropriate strains, followed by multiple rounds of backcrossing. The strains used here were considered congenic. Some mutants, such as *tup1*Δ (YTH3922), were created by one-step homologous recombination as previously described [54]. Primers flanking the *TUP1 *ORF by 500 basepairs were used in PCR reaction with genomic DNA from YTH1449 as template. Colonies that grew on Geneticin (G418) were confirmed by PCR. *GCN5 *was TAP-tagged on the C-terminus using one-step homologous recombination. Primers designed to flank the *GCN5 *stop codon by 500 basepairs on either side were used in PRC reactions with genomic DNA isolated from YTH3864 as template. PCR fragments were then transformed into YTH1235 cells. Colonies that formed on SD-his plates were confirmed by PCR and Western analyses. Plasmids and sources used in this study are provided in Table 2. Yeast and *E. coli *transformations were done according to published procedures [54]. Overexpression from the *CUP1 *promoter was accomplished by adding 0.4 mM CuSO_4 _to liquid growth media for 3 hours. Spot dilutions were performed by determining the OD_600 _of overnight cultures and then diluting the cells to 5 × 10^7^/ml. Ten-fold serial dilutions were prepared, with 3 μl volumes of each dilution spotted onto the appropriate media and incubated at a variety of temperatures. Northerns and Westerns were performed as described previously [54,60]. Primers used in the Northern analyses are shown in Table 3. Rabbit polyclonal anti-H3K9^Ac ^(Upstate Biotechnology), rabbit monoclonal anti-H3K14^Ac ^(Abcam), rabbit polyclonal anti-H3 (Abcam), rabbit polyclonal anti-HA (Abcam), and rabbit polyclonal anti-GST (Abcam) were used at 1:4000. Rabbit polyclonal anti-Clb2 (Santa Cruz; Y-180) and mouse monoclonal anti-ubiquitin (Cell Signalling Technology; P4D1) were used at 1:2000. The TAP antibody (Open Biosystems) was used at a dilution of 1:1000. Mouse monoclonal anti-GAPDH (Sigma) was used at a dilution of 1:20,000. Horseradish peroxidase (HRP)-conjugated secondary antibodies were used at a dilution of 1:20,000 for GAPDH, and for all other antibodies 1:10,000, and detected by enhanced chemiluminescene (PerkinElmer).

**Table 1:** Yeast strains used in this study

Strains	Relevant genotype	Source
YTH5	MATα *ade2 his3*Δ*200 lys2*Δ*201 ura 3-52*	[54]
YTH1235	MAT**a ***ade2 his3*Δ*200 lys2*Δ*201 ura3-52*	[60]
YTH1449	MAT**a*** his3*Δ*1 leu2*Δ* met15*Δ* ura3*Δ* tup1*Δ*::kanMX6*	ResGen
YTH1450	MAT**a*** his3*Δ1 *leu2*Δ* met15*Δ* ura3*Δ* ssn6*Δ*::kanMX6*	ResGen
YTH1235	MAT**a ***ade2 his3*Δ*200 lys2*Δ*201 ura3-52*	[60]
YTH1529	MAT(?) *ade2 his3 leu2 lys2(?) ura3 apc5^CA^-PA::His5^+ ^tup1*Δ*::kanMX6*	This study
YTH2305	MAT(?) *ade2 his3 leu2 lys2(?) ura3 hda1*Δ*::kanMX6*	[57]
YTH2306	MAT(?) *ade2 his3 leu2 lys2(?) ura3 apc5^CA^-PA::His5^+ ^hda1*Δ*::kanMX6*	[57]
YTH3393	MAT(?) *ade2 his3 leu2 lys2(?) ura3 gcn5*Δ*::kanMX6*	This study
YTH3395	MAT(?) *ade2 his3 leu2 lys2(?) ura3 apc5^CA^-PA::His5^+ ^gcn5*Δ*::kanMX6*	This study
YTH3477	MAT(?) *ade2 his3 leu2 lys2(?) ura3 gcn5*Δ*::kanMX6 hda1*Δ*::kanMX6*	This study
YTH3480	MAT(?) *ade2 his3 leu2 lys2(?) ura3 apc5^CA^-PA::His5^+ ^gcn5*Δ*::kanMX6 hda1*Δ*::kanMX6*	This study
YTH3638	MAT**a ***his3*Δ*1 leu2*Δ* met15 *Δ*ura3*Δ *rpn10*Δ::*kanMX6*	ResGen
YTH3864	MAT**a*** his3*Δ*1 leu2*Δ* met15*Δ* ura3*Δ* GCN5-TAP::HIS3*	ResGen
YTH3883	as YTH1235, with *GCN5-TAP::HIS3*	[57]
YTH3922	as YTH5, with *tup1*Δ*::kanMX6*	This study
YTH3923	as YTH5, with *ssn6*Δ*::kanMX6*	This study
YTH4006	MAT(?) *ade2 his3 leu2 lys2(?) ura3 gcn5*Δ*::kanMX6 ssn6*Δ*::kanMX6*	This study
YTH4010	MAT(?) *ade2 his3 leu2 lys2(?) ura3 gcn5*Δ*::kanMX6 tup1*Δ*::kanMX6 *	This study
YTH4379	MAT**a*** his3*Δ*1 leu2*Δ *met15*Δ* ura3*Δ* APC5*-*TAP*::*HIS3 rpn10*Δ::*kanMX6*	This study

? denotes marker not determined.

**Table 2:** Plasmids used in this study

Plasmid name	Markers/Integrated genes	Source
YCp50	*URA3 CEN ARS*	[54]
*GAL_pro_*-*APC5-HA*	*2μ GAL10_pro_-APC5-HA URA3*	[57]
*GAL_pro_*-*GCN5-HA*	*2μ GAL10_pro_-GCN5-HA URA3*	[57]
*GAL_pro_*-*HDA1-HA*	*2μ GAL10_pro_-HDA1-HA URA3*	W. Xiao/ResGen
*GAL_pro_*-*HPA2-HA*	*2μ GAL10_pro_-HDA1-HA URA3*	W. Xiao/ResGen
pGEX4T1-*GST-TUP1*	*2μ CUP1_pro_-TUP1 URA3*	ExClone/Clontech
pGEX4T1-*GST-SSN6*	*2μ CUP1_pro_-TUP1 URA3*	ExClone/Clontech

**Table 3:** Primers generated for the Northern analysis

Gene	Forward primer	Reverse primer
*CDC20*	5'-TGCCAGAAAGCTCTAGAG	5'-AACGAGAAGAGTATGCCG
*PDS1*	5'-TGATGCCAGCTAACGAAG	5'-TTGTGGTAAGTCTGCATC
*BCY1*	5'-AATCGCAAGCCGAATTGC	5'-TGGGTCCTCGTTCACAAAG
*MAD2*	5'-AGGGTTCAACAAGGACAG	5'-GCACATTGAAGGACCATC
*RDN1*	5'-GGTGGAGTGATTTGTCTG	5'-ACGACGGAGTTTCACAAG

### Reverse transcriptase PCR (rtPCR)

Total RNA was treated with RNase-free DNase (Fermentas Life Sciences) following the manufacturer's recommendations. 1-5 μg of total RNA was used for cDNA synthesis using an oligo(dT) primer and M-MLV reverse transcriptase (Fermentas). RNA was incubated at 70°C for 10 minutes prior to the reverse transcriptase reaction. Finally, 1 μl of each cDNA sample was used as template in PCR reactions with the primers described in Table 4 to amplify each of the target messages. To determine the PCR linear range for each message, 50 μl PCR reactions were prepared using WT cDNA with each primer set (Table 3). 5 μl of each reaction was removed every 2 cycles, analyzed using 1% agarose gel electrophoresis, and stained with ethidium bromide (data not shown). The gel was scanned and ImageJ was used to determine the mid-linear range cycle for each reaction. Subsequent rtPCR reactions were set up to cycle only to the predetermined mid-linear range. Primers that amplified the noncoding 18S rRNA *RDN37-2*, which is within the *RDN1 *locus (referred to as *RDN1*), were designed to generate a fragment for use as a control in Northerns and rtPCR.

**Table 4:** Primers generated for the ChIP analysis

Gene	Forward primer	Reverse primer
*CDC20 *	5'-CCGAAAGAGGCAAAACGT	5'-TCTCTAGAGCTTTCTGGC
*PDS1*	5'-CACTATCACTTTCCGTGC	5'-CTTCGTTAGCTGGCATCA
*BCY1*	5'-GCAGAAGCCATAAGCTGA	5'-GGGCAAAGAAGATACCATC
*MAD2*	5'-GCCATGCTGTTTAATGTGGC	5'-AAATGTGCTATTGGGCCC
*RDN1*	5'-TATGGTATGGTGACGGAG	5'- CCACCTATTCCCTCTTGC

### Chromatin immunoprecipitation (ChIP)

ChIP was performed essentially as described elsewhere [82,83] with the following modifications: DNA fragment size achieved by sonication was 500-1000 bp, and 100 μg of protein lysate was used for each IP. Protein concentration was determined by a Bradford protein assay. 5 μg of ChIP grade rabbit polyclonal anti-acetyl-H3K9/14 (Upstate Biotechnology), rabbit polyclonal anti-H3 (Abcam), rabbit polyclonal HA antibody (Abcam), and rabbit polyclonal GST antibody (Abcam) were used for IP. One-tenth of the total volume of lysate was used as input for each sample. Sequential ChIP was performed as previously described [84]. In sequential ChIP experiements, the immune complexes were eluted by incubation for 30 minutes at 37°C in 10 mM DTT. After centrifugation, the supernatant was diluted 25 times with ChIP dilution buffer (1% Triton X-100, 2 mM EDTA, 150 mM NaCl, 20 mM Tris-HCl [pH 8.1]) and subjected again to ChIP using a different antibody. In this experiment, HA antibody was applied first, followed by GST antibody. Cross-linking of the immune complex was reversed by adding NaCl to a final concentration of 0.3 M and incubated overnight at 65°C. Samples were treated first with 1 μg/μl RNaseA (Millipore [formerly Upstate]) for 30 minutes at 37°C, followed by 1 μg/μl proteinase K (Millipore [formerly Upstate]) at 45°C for 1 hour. DNA was purified by chromatography on QIAquick columns, and eluted with elution buffer (PCR purification kit, Qiagen). PCR was performed for semiquantitative determination by standard end point PCR. 1 μl DNA was used for PCR, and the reaction continued to the predetermined mid-linear range for each primer set. The end point PCR product was resolved on a 1% agarose gel and visualized by ethidium bromide. Two independent experiments were performed for each ChIP. The gel bands from each experiment were analyzed by ImageJ, and the means and standard error were plotted for graphical representation. For time course experiments, 200 ml cultures were induced at a final concentration of 4% galactose. Samples (20 ml) were immediately removed, and again after 1, 3 and 5 hours. The 20 ml samples were in duplicate for Western and ChIP analysis.

## Competing interests

The authors declare that they have no competing interests.

## Authors' contributions

AI conducted the vast majority of the experiments described in this manuscript. ELT discovered the interaction between *apc5^CA^*, *gcn5*Δ and *hda1*Δ while performing the genetic screen designed to identify HAT and HDAC mutants that genetically interact with *apc5^CA ^*[57]. ELT, MEM and JM contributed to work demonstrating accumulation of Gcn5 and Elp3 in ubiquitin compromised strains. TAAH designed the study, supervised the work, and wrote the manuscript. All authors approve the final manuscript.
